# Trends in Dental Medication Prescribing in Australia during the COVID-19 Pandemic

**DOI:** 10.1177/2380084420986766

**Published:** 2021-01-10

**Authors:** M. Mian, L. Teoh, M. Hopcraft

**Affiliations:** 1 Oral and Maxillofacial Surgery Unit, Royal Dental Hospital of Melbourne, Carlton, Victoria, Australia; 2 Melbourne Medical School, University of Melbourne, Victoria, Australia; 3 Melbourne Dental School, University of Melbourne, Victoria, Australia

**Keywords:** coronavirus, SARS-CoV-2, prescription drugs, prescription drug monitoring programs, antimicrobials, opioid analgesics

## Abstract

**Introduction:**

The coronavirus disease 2019 (COVID-19) pandemic and subsequent restrictions on dental services have had a significant impact on the provision of dental care in Australia and around the world.

**Objectives:**

To investigate the impact of COVID-19 on medications prescribed by dentists under the Australian Pharmaceutical Benefits Scheme (PBS).

**Methods:**

Data on the number of dental prescriptions dispensed for all medications listed on the PBS Dental Schedule, from January 2019 to June 2020, were extracted from publicly available data sets. Analysis of prescription trends was performed for 1) total medications, 2) each major medication class, and 3) individual medications. The number of prescriptions dispensed in each month from January 2020 to June 2020 was compared to the same month in 2019 to determine the relative (percentage) change, and *z* statistics were used to determine whether changes were statistically significant.

**Results:**

There was a significant decrease in dental prescriptions in April 2020 compared to April 2019 (14,785, 18%; *P* < 0.05). Decreases in prescriptions for antibiotics (10,512, 16%; *P* < 0.05) and opioid analgesics (3,129, 18%; *P* < 0.05) were smaller compared to other major medication classes. There was a significant increase in June 2020, compared with June 2019, for prescriptions of amoxicillin with clavulanic acid (4,903, 20%; *P* < 0.05), tramadol (89, 46%; *P* < 0.05), and oxycodone (381, 73%; *P* < 0.05).

**Conclusion:**

Dental service restrictions during COVID-19 likely drove an unmet need for routine dental treatment, which had significant implications for public oral health following easing of restrictions. During the initial surge and subsequent lockdown, antibiotics and opioid analgesics may have been used an as alternative to routine operative treatment. Continued professional guidance is required to ensure dental prescribing remains evidence based during the pandemic period.

**Knowledge Transfer Statement:**

The COVID-19 pandemic and subsequent restrictions on dental practice have had a profound impact on the provision of dental care in Australia and elsewhere in the world. In this context, population-level medication surveillance is important to identify and respond to changes in prescribing patterns that have arisen due to COVID-19 and restrictions on the provision of dental care. This research is particularly important for governments, regulators, and professional associations to ensure therapeutic guidelines and recommendations during the pandemic period remain relevant and evidence based.

## Introduction

Severe acute respiratory syndrome coronavirus 2 (SARS-CoV-2), which causes coronavirus disease 2019 (COVID-19), was first reported to the World Health Organization (WHO) in December 2019, following clusters of severe respiratory illness in Wuhan, China (WHO 2020a). The outbreak was declared an international public health emergency by the WHO on January 30, 2020, and a pandemic on March 11, 2020. As of October 7, 2020, there have been over 35 million confirmed cases of COVID-19 internationally, resulting in over 1 million deaths (WHO 2020b). Australia’s first case of COVID-19 was identified on January 25, 2020. An increase in national cases led to a range of coordinated state and federal measures designed to reduce disease transmission, beginning on March 23, 2020. This resulted in initial successful suppression, with community transmission virtually eliminated by late April. However, there has been a recent resurgence in cases in Victoria from July.

In this context, the dental profession has adapted to the changing risks of COVID-19 in Australia. In March 2020, the Australian Dental Association (ADA) developed a framework of practice restrictions to help guide the response of the profession (ADA 2020). This has been used by the Australian Health Protection Principal Committee (AHPPC), comprising all state and territory chief health officers, to recommend levels of service restriction based on the risk of transmission in the dental practice setting. On March 25, 2020, the AHPPC recommended that all dental practices implement level 3 restrictions, directing dentists to defer all routine dental care and limit aerosol-generating procedures to emergency treatment only. This recommendation was downgraded to level 2 on April 26, 2020, due to decreasing national COVID-19 cases. Level 2 restrictions direct dentists to defer aerosol-generating procedures or minimize contaminated aerosol generation through the use of a rubber dam. Continued COVID-19 suppression resulted in restrictions to be again downgraded on May 11, 2020, to level 1, allowing for routine dental care in patients without epidemiological or clinical risk factors for COVID-19.

Dental services, including the provision of dental medications, have adapted to the ever-evolving health and economic challenges posed by the COVID-19 pandemic. During this period, provision of dental treatment has fallen significantly, with estimates of up to a 41% reduction in publicly subsidized dental services provided to children from March to June 2020 (Hopcraft and Farmer 2020). Concurrently, there have been reports from Australia and elsewhere in the world of increasing rates of dentist-prescribed antibiotics and other medications to meet oral health needs, while minimizing the risk of COVID-19 transmission (Wordley et al. 2020). Formal guidelines for dentists in the United Kingdom recommended a triage service based on the principles of “advice, analgesia and antibiotics” (National Health Service 2020). The ADA COVID-19 guidelines also recommend the use of analgesics and antimicrobials where clinically indicated in situations where dental treatment is not possible (ADA 2020).

In Australia, medications, including those prescribed by dentists, are subsidized by the Australian government under the Pharmaceutical Benefits Scheme (PBS). The PBS accounts for approximately 75% of all dispensed medications in Australia (Mellish et al. 2015). Data on the number of medications prescribed under the PBS for general and concessional patients (including medications costed under the copayment amount) are collected and made publicly available (Australian Government Department of Health 2020a).

In the current COVID-19 landscape, population-level medication surveillance is important to identify and respond to changes in dental treatment and prescribing patterns that may have harmful effects on public health and oral health. This is part of the broader research agenda, outlined by the American Association for Dental Research, to better understand how COVID-19 is changing dental practice around the world (Herzberg 2020). Thus, the aim of this study was to investigate prescribing trends of dental medications listed on the dental PBS in Australia during the COVID-19 pandemic.

## Methods

### Data Source

Each medication listed under the PBS Dental Schedule has a unique code, and each dentist and dental specialist holds a dental prescriber number. These codes and prescriber numbers indicate dental prescriptions on the dispensed medications database, which is available on the PBS website (Australian Government Department of Health 2020a, 2020b). Medications prescribed under the PBS Dental Schedule must be for dental treatment to qualify for the benefit. This study analyzed monthly data on the number of prescriptions dispensed for all medications listed on the PBS Dental Schedule, from January 2019 to June 2020 (inclusive). This included prescriptions for all major classes of medications prescribed by Australian dentists under the PBS, namely, antibiotics, opioid analgesics, nonopioid analgesics, benzodiazepines, antiepileptics, antiemetics, antifungals, topical and injectable corticosteroids, and medicines for medical emergencies and conscious sedation. As all data were publicly accessible, ethical approval was not required.

### Data Analysis

Analysis of monthly prescriptions was performed for 1) total medications, 2) each major medication class, and 3) individual medications. The number of prescriptions dispensed in each month from January 2020 to June 2020 was compared to the same month in 2019 to determine absolute and percentage differences. This comparison accounted for seasonal and holiday-related changes, and *z*-statistics were used to determine whether differences were significant by comparing monthly prescriptions from January 2020 to June 2020 to the 2019 monthly mean. A *z* score of ±1.96, corresponding to a *P* value of <0.05, was considered statistically significant. The Shapiro-Wilk test for normality was used to confirm that numbers of monthly prescriptions for 1) total medications, 2) each major medication class, and 3) individual medications over 2019 were normally distributed. Data were analyzed using SciStat (MedCalc Software).

## Results

The number of prescriptions dispensed in each month from January 2020 to June 2020 for the most common medications on the PBS Dental Schedule is shown in the Table. The Shapiro-Wilk test confirmed that 2019 monthly prescriptions for 1) total medications, 2) each major medication class, and 3) individual medications were normally distributed. Data for all individual medications can be found in the Appendix Table.

**Table.** Summary of Dental PBS Prescriptions from January 2020 to June 2020.

	2019 Mean	January		February		March		April		May		June	
Characteristic		*N*	%	*N*	%	*N*	%	*N*	%	*N*	%	*N*	%
Antibiotics													
Amoxicillin	44,199	40,476	100	40,994	97	45,640	104	33,959^a^	80	40,782	86	47,030	112
Amoxicillin + clavulanic acid	8,591	8,021	106	8,008	102	9,573	112	7,986	97	8,959	98	9,945^a^	120
Cephalexin	1,971	1,707	94	1,896	99	2,033	100	1,383^a^	70	1,776	84	2,064	109
Clindamycin	3,286	2,839	94	2,963	94	3,357	99	2,378^a^	75	2,945	82	3,243	106
Metronidazole	9,190	8,422	95	8,584	99	9,630	106	8,600	99	9,261	95	9,992	116
Total antibiotics	68,984	62,825	99	63,852	97	71,866	105	55,529^a^	84	65,077	88	72,365	110
Opioid analgesics													
Codeine with paracetamol	17,866	16,354	102	16,418	99	17,929	103	14,060^a^	82	17,335	90	19,508	113
Oxycodone	514	604^a^	119	640^a^	119	597^a^	106	431^a^	83	721^a^	128	900^a^	173
Tramadol	212	200	105	221	113	198	84	201	105	266^a^	111	283^a^	146
Total opioid analgesics	18,608	17,175	103	17,300	100	18,740	103	14,697^a^	82	18,341	92	20,715	116
Nonopioid analgesics													
Diclofenac	255	252	114	240	111	237	93	143^a^	58	201^a^	71	251	92
Ibuprofen	1,030	893^a^	92	926	94	898^a^	85	566^a^	55	747^a^	68	937	95
Naproxen	213	221	140	238	133	230	94	161	76	248	106	285^a^	159
Total nonopioid analgesics	1,610	1,475	102	1,504	100	1,473	88	927^a^	59	1,331^a^	78	1,580	103
Benzodiazepines													
Diazepam	897	886	115	922	109	979	110	505^a^	63	910	97	1,120^a^	126
Temazepam	144	145	113	123	78	155	103	70^a^	51	115^a^	76	166	110
Total benzodiazepines	1,073	1,065	116	1,088	104	1,169	109	593^a^	61	1,058	94	1,343^a^	125
Antifungals	223	183	110	230	88	228	97	92^a^	50	184	76	221	98
Antiemetics	50	34^a^	69	27^a^	59	38	79	14^a^	38	9^a^	20	31^a^	52
Antiepileptics	14	11	69	12	80	13	144	16	145	11	69	12	80

Total prescriptions per month (*N*) and percentage (%) compared to the same month in 2019.

a *P* < 0.05.

### Total Prescriptions

**Figure 1.**
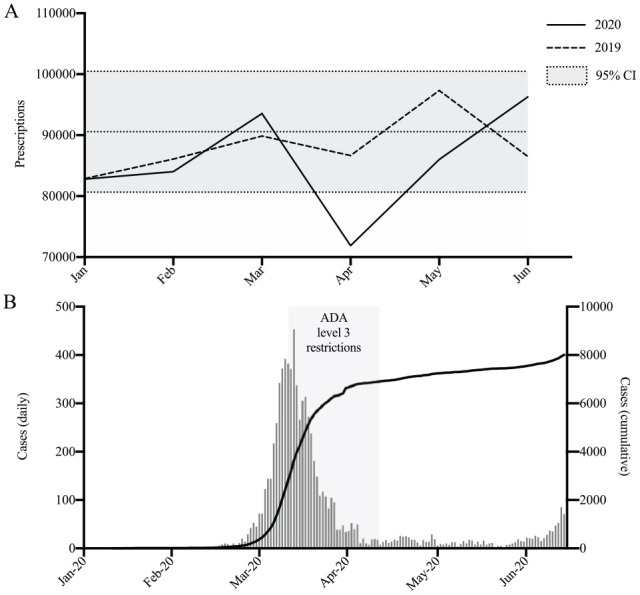
Dental prescriptions in Australia during COVID-19. (**A**) Total dental prescriptions from January to June 2019 and January to June 2020. The 2019 monthly mean and corresponding 95% confidence interval is indicated (gray area). (**B**) National COVID-19 cases per day (left axis) and cumulatively (right axis). Australian Dental Association (ADA) level 3 restrictions were implemented on March 25, 2020, and reduced to ADA level 2 restrictions on April 26, 2020.

The mean number of total monthly prescriptions for 2019 was 90,569 (95% confidence interval [CI], 80,654–100,484). Total prescriptions for each month of 2020 were as follows: January, 82,779; February, 84,020; March, 93,541; April, 71,872; May, 86,020; and June, 96,281. There was a decrease in total prescriptions in April 2020 compared to April 2019 (14,785, 18%; *P* < 0.05) (Figure 1).

### Antibiotics

**Figure 2.**
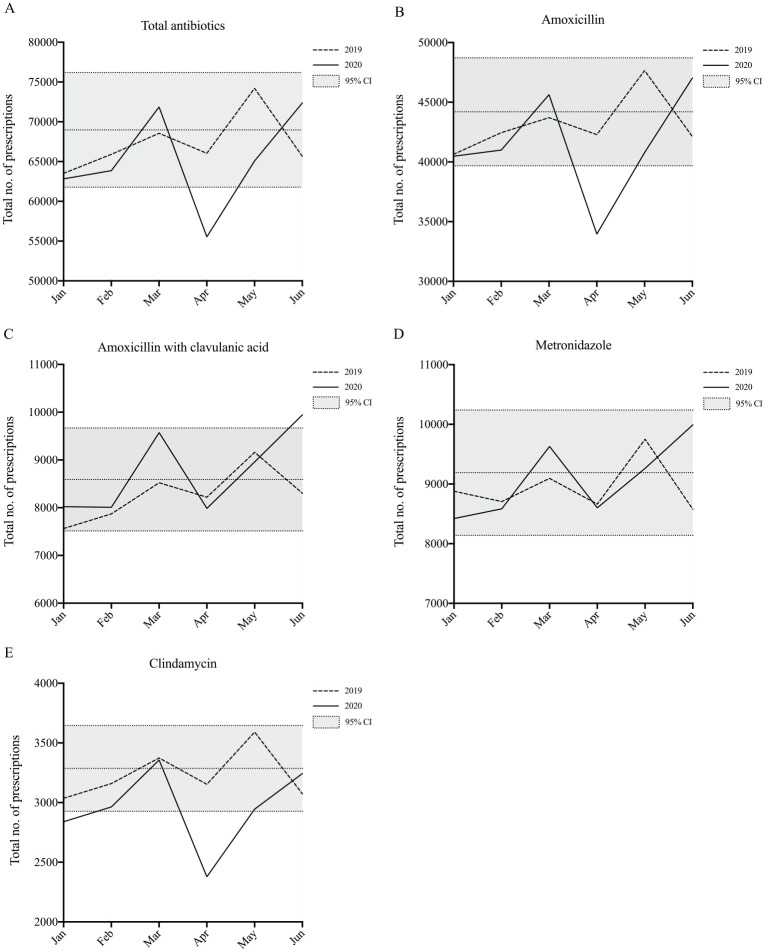
Antibiotic prescriptions from January to June 2019 and January to June 2020 are shown for (**A**) total antibiotics, (**B**) amoxicillin, (**C**) amoxicillin with clavulanic acid, (**D**) metronidazole, and (**E**) clindamycin. The 2019 monthly mean and corresponding 95% confidence interval is indicated (gray area).

Antibiotics were the most commonly prescribed drug class, representing approximately 76% of total PBS dental prescriptions dispensed from January to June 2020. The most commonly prescribed antibiotic was amoxicillin, representing 64% of total antibiotic prescriptions. This was followed by metronidazole (14%), amoxicillin with clavulanic acid (13%), clindamycin (5%), and cephalexin (3%). There was a decrease in April 2020, compared with April 2019, for total prescriptions of antibiotics (10,512, 16%; *P* < 0.05). There was a decrease in April 2020, compared with April 2019, for prescriptions of amoxicillin (8,326, 20%), cephalexin (602, 30%), and clindamycin (775, 25%; *P* < 0.05). There was an increase in June 2020, compared with June 2019, for prescriptions of amoxicillin with clavulanic acid (4,903, 20%; *P* < 0.05) (Figure 2).

### Opioid Analgesics

**Figure 3.**
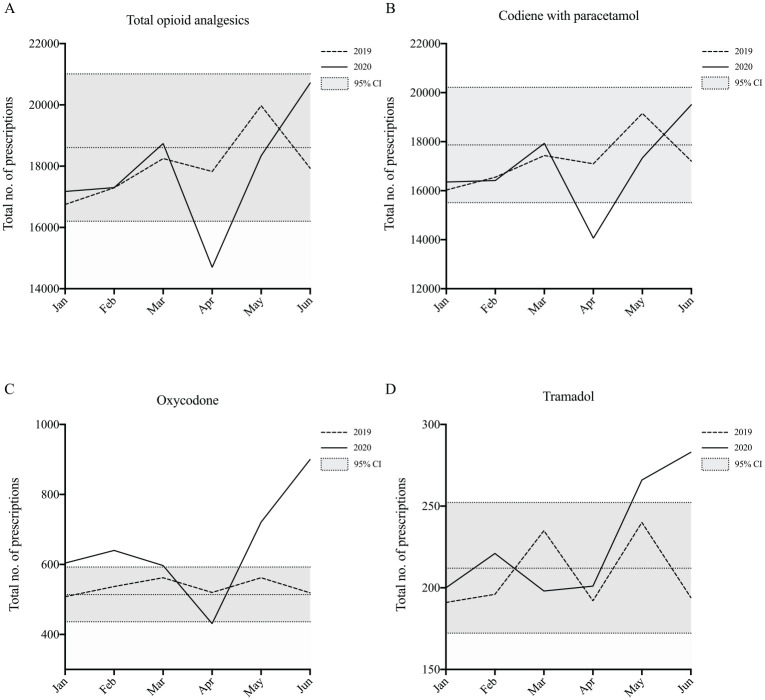
Opioid analgesic prescriptions from January to June 2019 and January to June 2020 are shown for (**A**) total opioid analgesics, (**B**) codeine with paracetamol, (**C**) oxycodone, and (**D**) tramadol. The 2019 monthly mean and corresponding 95% confidence interval is indicated (gray area).

Opioid analgesics were the second most commonly prescribed drug class, representing approximately 21% of total PBS dental prescriptions dispensed over 2020. The most commonly prescribed opioid analgesic was codeine with paracetamol, representing 95% of total opioid analgesic prescriptions. This was followed by oxycodone (4%) and tramadol (1%). There was a decrease in April 2020, compared with April 2019, for total prescriptions of opioid analgesics (3,129, 18%; *P* < 0.05). Within the opioid analgesic class, there was a decrease in April 2020, compared with April 2019, for prescriptions of codeine with paracetamol (3037, 18%) and oxycodone (89, 17%; *P* < 0.05). There was an increase in May 2020, compared with May 2019, for prescriptions of tramadol (26, 11%; *P* < 0.05). There was also an increase in June 2020, compared with June 2019, for prescriptions of tramadol (89, 46%; *P* < 0.05). There was an increase in every month of 2020, except April, compared to equivalent months in 2019, for prescriptions of oxycodone (mean = 134, mean = 22%; *P* < 0.05) (Figure 3).

### Nonopioid Analgesics

Nonopioid analgesics represented approximately 2% of total PBS dental prescriptions dispensed over 2020. The most commonly prescribed nonopioid analgesic was ibuprofen, representing 60% of total nonopioid analgesic prescriptions. This was followed by naproxen (16%) and diclofenac (16%). There was a decrease in April 2020, compared with April 2019, for total prescriptions of nonopioid analgesics (650, 41%; *P* < 0.05). There was also decrease in May 2020, compared with May 2019, for total prescriptions of nonopioid analgesics (384, 22%;*P* < 0.05). Within the nonopioid analgesic class, there was a decrease in March 2020, compared with March 2019, for prescriptions of ibuprofen (157, 15%; *P* < 0.05). There was a decrease in April 2020, compared with April 2019, for prescriptions of ibuprofen (456, 45%) and diclofenac (102, 42%; *P* < 0.05). There was also a decrease in May 2020, compared with May 2019, for prescriptions of ibuprofen (353, 32%) and diclofenac (81, 29%; *P* < 0.05). There was an increase in June 2020, compared with June 2019, for prescriptions of naproxen (52, 59%; *P* < 0.05).

### Benzodiazepines

Benzodiazepines represented approximately 1% of total PBS dental prescriptions dispensed over 2020. The most commonly prescribed benzodiazepines were diazepam, representing 85% of total benzodiazepine prescriptions, followed by temazepam (12%). There was a decrease in April 2020, compared with April 2019, for total prescriptions of benzodiazepines (378, 39%; *P* < 0.05). There was an increase in June 2020, compared with June 2019, for total prescriptions of benzodiazepines (267, 25%; *P* < 0.05). Within the benzodiazepine class, there was a decrease in April 2020, compared with April 2019, for prescriptions of diazepam (293, 37%) and temazepam (67, 49%). There was a decrease in May 2020, compared with May 2019, for prescriptions of temazepam (36, 24%). There was an increase in June 2020, compared with June 2019, for diazepam (228, 26%; *P* < 0.05).

### Other Medications

Prescriptions for all other medications, including antifungals, antiepileptics, antiemetics, corticosteroids, and medicines for medical emergencies and conscious sedation, in total, represented less than 1% of dental prescriptions in 2020. There was a decrease in April 2020, compared with April 2019, for total prescriptions of antifungals (93, 47%) and antiemetics (23, 62%; *P* < 0.05). There was a decrease in May 2020, compared with May 2019, for total prescriptions of antiemetics (36, 80%; *P* < 0.05). There was also a decrease in June 2020, compared with June 2019, for total prescriptions of antiemetics (29, 48%;*P* < 0.05).

## Discussion

The COVID-19 pandemic continues to have a significant impact across virtually all aspects of health and the economy, including the provision of dental care. Dental staff and patients are presumed to be at a high risk of infection because of the close contact with the patient’s oral cavity, as well as the presence of the virus in bioaerosols generated by rotary instrumentation (Ren et al. 2020). In Australia, restrictions on dental services were implemented to reduce the risk of transmission in the dental practice setting. These have aimed to provide a proportional response based on the changing risk of viral transmission. The highest level of restrictions was implemented nationally from March 25, 2020, to April 23, 2020, and effectively restricted dental treatment to emergency-only procedures. Dentists and dental specialists have adapted to this evolving landscape. This study aimed to investigate changes in prescribing trends of dental medications listed on the dental PBS in Australia during the COVID-19 pandemic. To our knowledge, this is the first study to investigate the population-level effects of COVID-19 on dental prescribing anywhere in the world.

There was an 18% decrease in total prescriptions during April 2020. This period largely corresponded to stage 3 national lockdowns and ADA level 3 restrictions, both of which are likely to have contributed to reduced total dental visits. This is reflected in data from the Child Dental Benefits Schedule, which showed an 80% reduction in total dental services during this time (Hopcraft and Farmer 2020). Total prescriptions partially recovered in May and were increased by 11% in June compared to 2019, although this increase was not statistically significant. The total number of prescriptions from March to June 2020 was only 12,633 (3%) fewer than the same period in 2019. During the national lockdown, it is likely that there was a buildup of demand for dental services, which were initially deferred as a result of dental restrictions and then provided following control of COVID-19 and easing of restrictions in Australia. Concerningly, there was a significant increase in the use of broad-spectrum antibiotics and opioid analgesics in June 2020 compared to June 2019. These increases also likely reflect the increased demand for dental services following the national lockdown. However, given that the main indications for these medications in dentistry are severe spreading infection and severe acute pain, respectively (Oral and Dental Expert Group 2019), it is possible that these increases may also reflect clinical deterioration of patients with existing disease as a result of delayed treatment. Continued deferral of routine dental treatment during the pandemic is likely to have significant public oral health implications. This includes increased rates of untreated dental decay and periodontal disease, resulting in progressive suffering and poorer dental outcomes over time (Ellershaw and Spencer 2011). The oral health needs of populations must be balanced against the risk of COVID-19 transmission in the dental setting. The WHO continues to recommend against resumption of routine dental care where there is still significant community transmission (WHO 2020c). However, where there is sufficient disease suppression, it may be reasonable to provide routine dental care with appropriate precautions (FDI World Dental Federation 2020).

There were smaller decreases for prescriptions of antibiotics and opioid analgesics in the April and March periods, compared to other major classes of medications. As population-level data are not available for adult dental services, it is difficult to estimate the number of antibiotic or opioid analgesic prescriptions per dental visit. However, if decreases of 80% and 50% of child dental visits in April and May, respectively (Hopcraft and Farmer 2020), are somewhat reflective of the total reduction in adult visits, then these findings would suggest a significantly *increased* rate of antibiotic and opioid prescriptions per visit. There are 2 possible explanations. First, antibiotics and opioid analgesics are generally more commonly prescribed for high-acuity dental conditions (Oral and Dental Expert Group 2019), which are more likely to require emergency treatment and therefore less likely to be affected by restrictions on routine dental care. Second, it is possible that prescriptions for antibiotics and opioid analgesics were used as an alternative to active surgical treatment to reduce bioaerosol generation. This is particularly likely given the shortage of face masks in the early pandemic period due to decreased international supply and increased utilization by other health care services (Mahase 2020). In the United Kingdom, where professional guidelines were based on the triage principles of “advice, analgesia, and antimicrobials,” rates of antimicrobial prescribing increased, including in cases where there is no evidence for such medications (Grossman et al. 2020). While mandatory ADA level 3 restrictions guided dentists to address acute dental pain with appropriate endodontic or restorative treatment, this did not stop individual dentists or dental practices from implementing their own voluntary restrictions on services.

In contrast to other antibiotics, prescriptions for metronidazole and amoxicillin with clavulanic acid during lockdown period were similar to levels in 2019. The most common indication for these antibiotics in the dental setting is for management of severe odontogenic infections (Oral and Dental Expert Group 2019). In these instances, patients are more likely to seek dental treatment, and correspondingly, dentists are more likely to provide treatment, meaning COVID-19 and restrictions on dental practice would have less of an effect compared to other medications with nonurgent indications. In addition, a number of previous studies have suggested that dentists tend to provide a broader-spectrum antibiotic, such as amoxicillin with clavulanic acid, in cases where no operative treatment is undertaken (Palmer et al. 2000; Mainjot et al. 2009; Teoh et al. 2018). This is also likely to be a factor as dentists may be attempting to avoid bioaerosol generation and transmission of COVID-19 in the dental practice setting. Use of broad-spectrum antibiotics is also more likely to contribute to adverse drug events and increased antibiotic resistance in the community, which should also be borne in mind by prescribers (Teoh et al. 2018).

There was a significant increase in oxycodone prescriptions for every month of 2020, except April. This is likely to reflect changes to the national therapeutic guidelines published in December 2019, which recommend oxycodone as the preferred opioid analgesic for severe acute nociceptive dental pain. Reasons cited for the change include widespread experience with its use, fewer drug interactions than tramadol, and more predictable pharmacokinetics and greater efficacy than codeine (Oral and Dental Expert Group 2019). It is encouraging that dentists appear to have begun to transition to oxycodone, but codeine with paracetamol is still being prescribed at a rate 30 times higher than oxycodone, and use of tramadol also increased in May and June 2020 compared to 2019 levels. It is likely that more time and increased attention to the changes in the therapeutic guidelines are required for the professional transition to current best practice. The increase in opioid use in the postlockdown period is also concerning given that opioid dependence and addiction represent a major public health issue (Campbell et al. 2019). In this context, dentists may be inadvertently contributing to the misuse of pharmaceutical opioids through inappropriate prescribing. In patients without contraindications, nonsteroidal anti-inflammatory drugs are the most appropriate first-line analgesics (Oral and Dental Expert Group 2019). Further education about the appropriate use and limitations of opioids in general dental practice may be warranted (Teoh et al. 2020).

This study presents population-level data on the effect of COVID-19 on the prescription of dental medications in Australia. A number of limitations should be kept in mind when interpreting the results presented here. First, data presented here do not represent all dental prescribing in Australia. Medications not listed on the PBS Dental Schedule or prescriptions not satisfying PBS eligibility criteria are dispensed as private prescriptions. In addition, a pharmacist may choose to dispense a PBS-listed medication as a private prescription if it is cheaper for a patient. Also, medications prescribed to inpatients in public hospitals and medications prescribed by specialist oral and maxillofacial surgeons, who use their medical prescriber number, will not be included here. Furthermore, prescriptions may be used to help understand the amount and acuity of oral disease being managed by dentists. However, PBS data are not linked to dental service records and cannot be used to accurately infer the type of operative treatment being provided. Last, we note that date-of-supply data are finalized up to 6 mo after their initial publication on the PBS website (Mellish et al. 2015). Nonetheless, changes are usually very minor and unlikely to affect the overall results of this study. Moreover, given the rapidly evolving nature of the COVID-19 pandemic, the most contemporaneous data are needed to inform the decisions of profession moving forward.

## Conclusion

The COVID-19 pandemic continues to have a significant impact across all aspects of health and the economy, including the provision of dental care. This study investigated the trends in prescriptions for PBS dental medications in Australia over the COVID-19 pandemic. Total prescriptions during the lockdown period fell, reflecting a reduction in total dental visits. Use of antibiotics and opioid analgesics during this period remained relatively high, consistent with an increased use of these medications as an alternative to routine operative treatment to reduce COVID-19 transmission. Following easing of service restrictions, prescriptions for broad-spectrum antibiotics and opioid analgesics increased, which may reflect clinical deterioration as a result of delayed treatment during the lockdown period. Moving forward, the oral health needs of the public should be balanced against the risk of COVID-19 transmission in the dental setting. Continued professional guidance is required to ensure dental prescribing remains in line with current best evidence.

## Author Contributions

M. Mian, L. Teoh, contributed to conception, design, data acquisition, analysis, and interpretation, drafted and critically revised the manuscript; M. Hopcraft, contributed to conception, design, data analysis, and interpretation, drafted and critically revised the manuscript. All authors gave final approval and agree to be accountable for all aspects of the work.

## Supplemental Material

sj-pdf-1-jct-10.1177_2380084420986766 – Supplemental material for Trends in Dental Medication Prescribing in Australia during the COVID-19 Pandemic Click here for additional data file. Supplemental material, sj-pdf-1-jct-10.1177_2380084420986766 for Trends in Dental Medication Prescribing in Australia during the COVID-19 Pandemic by M. Mian, L. Teoh and M. Hopcraft in JDR Clinical & Translational Research
