# Supplementary material for: Trends in Dental Medication Prescribing in Australia during the COVID-19 Pandemic
Source: JDR Clin Trans Res. 2021 Jan 10;6(2):145–52. doi: 10.1177/2380084420986766 (PMC7803793; doi:10.1177/2380084420986766)
Supplement: sj-pdf-1-jct-10.1177_2380084420986766 – Supplemental material for Trends in Dental Medication Prescribing in Australia during the COVID-19 Pandemic [file sj-pdf-1-jct-10.1177_2380084420986766.pdf]

Appendix Table – Data for total medications, each major medication class and individual medications on the Australian Dental PBS from January 2019 to June 2020

	Jan 19	Feb 19	Mar 19	Apr 19	May 19	Jun 19	Jul 19	Aug 19	Sep 19	Oct 19	Nov 19	Dec 19	Jan 20	Feb 20	Mar 20	Apr 20	May 20	Jun 20
Total prescriptions	82869	86085	89852	86657	97332	86506	97107	97004	89565	95149	90727	87979	82779	84020	93541	71872	86020	96281
Antibiotics	63519	65911	68550	66041	74211	65657	73525	73602	68245	72326	68805	67419	62825	63852	71866	55529	65077	72365
Antiemetics	49	46	48	37	45	60	43	60	45	47	55	61	34	27	38	14	9	31
Antifungals	166	260	235	185	242	225	214	219	223	233	246	227	183	230	228	92	184	221
Benzodiazepines	921	1045	1070	971	1122	1076	1187	1222	1041	1156	1139	922	1065	1088	1169	593	1058	1343
Corticosteroids	1	3	6	2	1	1	3	1	5	5	1	4	3	1	4	1	1	2
Non-opioid analgesics	1442	1502	1680	1577	1715	1540	1686	1699	1591	1710	1643	1533	1475	1504	1473	927	1331	1580
Opioids	16749	17295	18248	17826	19975	17930	20423	20194	18387	19659	18810	17803	17175	17300	18740	14697	18341	20715
Other	22	23	15	18	21	17	26	7	28	13	28	10	19	18	23	19	19	24
Adrenaline	4	3	2	3	1	0	4	0	2	0	1	0	3	0	0	1	2	3
Amoxicillin	40633	42450	43712	42285	47659	42127	46689	47132	43808	46304	44336	43257	40476	40994	45640	33959	40782	47030
Amoxicillin + clavulanic acid	7563	7871	8523	8220	9160	8302	9292	9171	8462	8979	8694	8855	8021	8008	9573	7986	8959	9945
Amphotericin	162	247	225	180	230	216	200	196	215	224	234	216	178	216	217	87	181	219
Ampicillin	3	2	1	0	1	0	0	2	2	2	0	5	0	0	0	0	0	1
Atropine	0	0	0	0	0	0	0	0	1	0	0	0	0	0	0	0	0	0
Benzathine benzylpenicillin	0	0	1	2	1	0	0	0	0	0	0	0	0	0	0	0	1	0
Benzatropine	0	0	0	0	0	0	0	0	0	0	0	0	0	0	0	0	0	0
Benzdamine mouthwash	1	0	0	0	0	0	0	0	1	0	0	0	1	1	3	0	0	1
Benzylpenicillin	0	0	0	0	1	0	0	2	0	0	0	0	0	0	0	0	0	0
Betamethasone injection	0	1	2	1	0	0	2	0	2	2	0	4	1	1	1	0	1	1
Carbamazepine	16	15	9	11	16	15	18	6	17	11	26	7	11	12	13	16	11	12
Cefaclor	37	54	54	35	41	57	45	47	45	42	52	42	42	33	39	62	34	45
Cefotaxime	0	0	0	0	0	0	0	0	0	0	0	0	0	0	0	0	0	0
Cefuroxime	4	6	5	4	6	2	5	3	4	2	8	8	4	5	9	3	2	0
Cephalexin	1824	1913	2026	1985	2108	1889	2063	2115	1902	2050	1948	1824	1707	1896	2033	1383	1776	2064
Cephalotin	0	0	0	0	0	0	0	0	0	0	0	0	0	0	0	0	0	0

Clindamycin	3036	3159	3374	3153	3591	3073	3482	3480	3268	3409	3270	3140	2839	2963	3357	2378	2945	3243
Codeine	22	9	15	17	18	9	21	13	18	23	13	10	17	19	16	5	19	22
Codeine with paracetamol	16028	16553	17435	17097	19155	17207	19608	19432	17682	18920	18140	17131	16354	16418	17929	14060	17335	19508
Diazepam	772	847	886	798	942	892	988	1037	906	986	954	753	886	922	979	505	910	1120
Diclofenac	222	216	254	245	282	273	264	278	285	275	248	222	252	240	237	143	201	222
Dicloxacillin	1	2	0	1	0	0	3	5	3	2	3	1	1	4	5	3	2	1
Doxycycline	107	136	103	106	124	108	119	117	104	121	122	117	99	103	110	65	85	91
Erythromycin	577	668	719	649	738	611	729	720	660	720	621	619	530	561	627	458	512	608
Flucloxacillin	23	22	30	22	14	24	20	19	15	27	27	30	29	23	29	22	18	29
Glucagon	1	0	1	0	2	0	1	0	2	0	0	0	0	0	0	0	1	0
GTN	0	0	0	0	0	0	0	0	0	0	0	0	0	0	0	0	0	0
Hydrocortisone injection	0	0	1	0	0	0	0	0	1	0	0	0	0	0	2	0	0	0
Hydromorphone	0	0	0	0	0	0	0	0	0	0	0	0	0	1	0	0	0	0
Ibuprofen	967	984	1055	1022	1100	983	1075	1094	965	1102	1035	982	893	926	898	566	747	967
Indomethacin	5	5	7	8	7	8	7	10	6	10	11	20	10	11	13	11	10	5
Ketoprofen	11	13	15	12	11	14	7	8	6	10	6	6	11	9	8	6	12	11
Lincomycin	0	0	0	1	0	0	0	1	0	0	0	0	0	0	0	0	0	0
Methylprednisolone injection	1	1	1	0	1	0	0	1	2	2	1	0	2	0	1	1	0	0
Metoclopramide	30	33	36	29	28	38	31	38	27	31	37	37	16	11	16	7	5	17
Metronidazole	8879	8705	9094	8664	9751	8577	10102	9851	9095	9733	8933	8896	8422	8584	9630	8600	9261	9992
Morphine	0	0	1	0	0	1	0	1	0	0	0	0	0	1	0	0	0	2
Naloxone	0	0	0	0	0	0	0	0	0	0	0	0	1	0	0	0	0	0
Naproxen	158	179	245	212	233	179	222	209	233	235	245	202	221	238	230	161	248	158
Nitrazepam	5	5	10	8	5	9	4	8	5	5	8	5	5	6	5	7	5	7
Nystatin	4	13	10	5	12	9	14	23	8	9	12	11	5	14	11	5	3	2
Oxazepam	16	35	24	28	24	24	30	22	15	25	30	34	29	37	30	11	28	50
Oxycodone	508	537	562	520	562	519	571	515	468	486	472	453	604	640	597	431	721	900
Paracetamol	31	57	59	40	27	35	43	38	38	30	26	45	40	33	41	21	26	31
Phenoxymethylpenicillin	706	783	754	767	862	752	814	778	756	827	690	486	524	564	656	527	612	650
Piroxicam	48	48	45	38	55	48	68	62	58	48	72	56	48	47	46	19	87	48
Procaine penicillin	0	2	0	0	1	0	2	0	0	1	1	0	0	0	0	1	0	0

Prochlorperazine	19	13	12	8	17	22	12	22	18	16	18	24	18	16	22	7	4	14
Promethazine	0	0	0	0	0	1	0	0	0	0	0	0	0	0	0	0	0	0
Roxithromycin	87	104	112	112	122	103	120	125	87	81	80	109	103	91	122	58	70	82
Temazepam	128	158	150	137	151	151	165	155	115	140	147	130	145	123	155	70	115	166
Topical hydrocortisone	0	5	3	4	2	1	3	1	5	2	1	3	3	5	7	2	5	8
Tramadol	191	196	235	192	240	194	223	233	219	230	185	209	200	221	198	201	266	283
Triamcinolone injection	0	1	2	1	0	1	1	0	0	1	0	0	0	0	0	0	0	1
Trimethoprim + sulfamethoxazole	39	34	42	35	31	32	40	34	34	26	20	30	28	23	36	24	18	19
Vancomycin	0	0	0	0	0	0	0	0	0	0	0	0	0	0	0	0	0	0
